# Complete mitochondrial genome of brownstripe red snapper, *Lutjanus vitta* (Perciformes: Lutjanidae)

**DOI:** 10.1080/23802359.2018.1521309

**Published:** 2018-10-08

**Authors:** Sapto Andriyono, Jobaidul Alam, Dong Hee Kwak, Hyun-Woo Kim

**Affiliations:** a Interdisciplinary Program of Biomedical, Mechanical and Electrical Engineering, Pukyong National University, Busan, Republic of Korea;; b Department of Marine, Fisheries and Marine Faculty, Universitas Airlangga, East Java, Indonesia;; c Department of Marine Biology, Pukyong National University, Busan, Republic of Korea

**Keywords:** Next generation sequencing, *Lutjanus vitta*, mitogenome, snapper, Indonesia

## Abstract

The complete mitochondrial genome of the brownstripe red snapper, *Lutjanus vitta* was determined by MiSeq sequencing platform. The mitogenome of *L. vitta* (16,498 bp) encoded the typical 13 protein-coding genes, 22 tRNA genes, two rRNA genes, and two non-coding regions (origin of light strand replication; O_L_ and *D-Loop* control region; CR). Phylogenetic analysis based on the full mitochondrial genome sequences showed that *L. vitta* is most closely related to *L. russellii*. The complete mitogenome sequence of *L. vitta* would provide the information of the genetic biodiversity in *Lutjanus* fishes, which would be further used for their scientific management in Indonesian waters.

The brownstripe red snapper, *Lutjanus vitta* is one of the economically important marine fish species, which is widely distributed in the western Pacific and Indian Ocean (Iwatsuki et al. 1993; Salini et al. 2006). Since *L. vitta* uses the coral reef as their spawning and feeding ground (Freitas et al. 2011), a high degree of genetic variations are expected considering its wide distribution along with coral reefs. The genetic information of *L. vitta* should be supplemented to manage scientifically its resource for the sustainable use.

Full mitochondrial genome sequence of *L. vitta* was determined by next-generation sequencing (NGS) platform. The specimen was collected from the coastal water in Pekalongan, Central Java, Indonesia (6°51'45″ S 109°4'24″ E) and stored at Universitas Airlangga, Indonesia. Species identification of the specimen was confirmed by both its morphological characteristics and DNA sequence identity in the COI region (GenBank Accession number: EU600101). Mitochondrial DNA was extracted by the mitochondrial DNA isolation kit (Abcam, UK) according to the manufacturer’s protocol. Purified mitochondrial DNA was further fragmented into smaller sizes (∼350 bp) by Covaris M220 Focused-Ultrasonicator (Covaris Inc., Woburn, MA, USA). A library for sequencing was constructed by TruSeq® RNA library preparation kit V2 (Illumina Inc., San Diego, CA, USA) and its quality and the quantity was analyzed by 2100 Bioanalyzer (Agilent Technologies, Santa Clara, CA, USA). DNA sequencing was performed by Illumina MiSeq sequencer (Illumina Inc., San Diego, CA, USA) (2 × 300 bp pair ends).

The complete mitochondrial genome of *L. vitta* (GenBank Number: MH675887) was 16,498 bp in length, which consisted of 13 protein-coding genes, 22 tRNAs, two ribosomal RNAs (12S and 16S), and two non-coding regions (origin of light strand replication; O_L_ and D-Loop control region; CR). tRNA genes ranged from 69 bp to 76 bp in length. Eight tRNA genes were on L-strand while the other 14 tRNAs on H-strand formed the conserved three tRNA clusters (IQM, WANCY, and HSL) (Satoh et al. 2016). As a result of ARWEN (Laslett and Canbäck 2008), all the tRNAs were predicted to be folded into the typical clover-leaf secondary structures except for tRNA-*Ser*. Two non-coding regions, O_L_ and CR, were located between tRNA-*Asn* and tRNA-*Cys* at WANCY cluster and between tRNA-*Pro* and tRNA-*Phe*, respectively. Besides COX1 gene, which was initiated by GTG, all other protein-coding genes begin with typical ATG start codons and incomplete stop codons were identified in ND2, COX2, COX3, ND3, ND4, and Cyt b genes.

The phylogenetic tree of *L. vitta* was constructed with other 10 complete mitogenomes in Lutjanidae by MEGA7.0 program with minimum evolutionary (ME) algorithm (Kumar et al. 2016). Based on the full mitogenome sequences in the database, *L. vitta* was most closely related to *L. russellii* (NC010963) with 90% nucleotide sequence identity (Figure 1). As the result with COX1 genes, *L. vitta* showed the highest identity to *L. fulvus* (93%, EU502667) followed by *L. russellii* (91%) suggesting *L. fulvus* may be the most closely related species to *L. vitta* among currently known Lutjanidae species in database.

**Figure 1.**
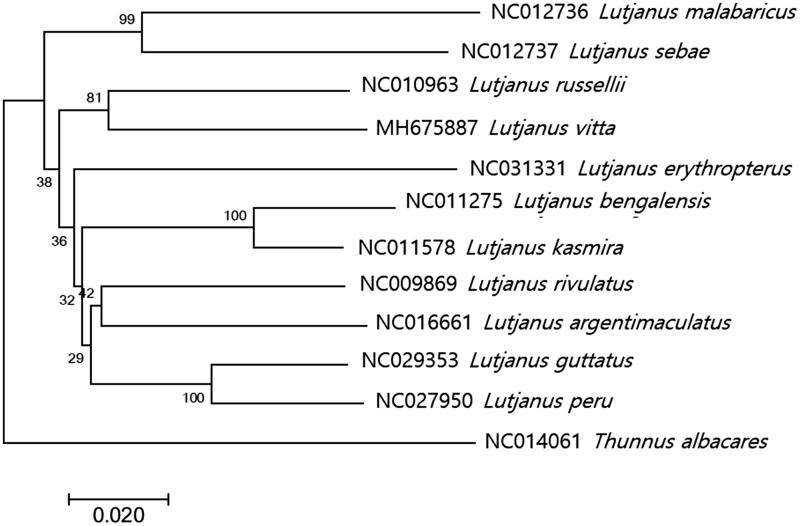
Phylogenetic tree of *Lutjanus vitta* within Lutjanidae. Phylogenetic tree of *Lutjanus vitta* complete genome was constructed by MEGA7 software with Minimum Evolution (ME) algorithm with 1000 bootstrap replications. GenBank Accession numbers were shown followed by each scientific name. The sequence data for phylogenetic analyses used in this study were as follows: *Lutjanus vitta* (MH675887), *Lutjanus russellii* (NC010963), *Lutjanus bengalensis* (NC011275), *Lutjanus kasmira* (NC011578), *Lutjanus rivulatus* (NC009869), *Lutjanus argentimaculatus* (NC016661), *Lutjanus peru* (NC027950), *Lutjanus guttatus* (NC029353), *Lutjanus erythropterus* (NC031331), *Lutjanus sebae* (NC012736), *Lutjanus malabaricus* (NC012736), and furthermore *Thunnus albacares* (NC014061) as an outgroup.
